# Selective growth of palladium and titanium dioxide nanostructures inside carbon nanotube membranes

**DOI:** 10.1186/1556-276X-7-342

**Published:** 2012-06-25

**Authors:** Samuel Hevia, Pía Homm, Andrea Cortes, Verónica Núñez, Claudia Contreras, Jenniffer Vera, Rodrigo Segura

**Affiliations:** 1 Departamento de Física, Pontificia Universidad Católica de Chile, Av. Vicuña Mackena, 4860, Santiago, Chile; 2 Departamento de Física, Universidad Técnica Federico Santa María, Av. España, 1680, Valparaíso, Chile; 3 Departamento de Química y Bioquímica, Facultad de Ciencias, Universidad de Valparaíso, Av. Gran Bretaña, 1111, Valparaíso, Chile; 4 Programa Conjunto de Doctorado en Ciencias Mención Química, Universidad Técnica Federico Santa María - Universidad de Valparaíso, Valparaíso, Chile

**Keywords:** carbon nanotubes, nanohybrids, palladium nanoparticles, titanium oxide, 81.07.-b, 81.15.Gh, 81.07.De

## Abstract

Hybrid nanostructured arrays based on carbon nanotubes (CNT) and palladium or titanium dioxide materials have been synthesized using self-supported and silicon-supported anodized aluminum oxide (AAO) as nanoporous template. It is well demonstrated that carbon nanotubes can be grown using these membranes and hydrocarbon precursors that decompose at temperatures closer to 600°C without the use of a metal catalyst. In this process, carbonic fragments condensate to form stacked graphitic sheets, which adopt the shape of the pores, yielding from these moulds' multi-walled carbon nanotubes. After this process, the ends of the tubes remain open and accessible to other substances, whereas the outer walls are protected by the alumina. Taking advantage of this fact, we have performed the synthesis of palladium and titanium dioxide nanostructures selectively inside carbon nanotubes using these CNT-AAO membranes as nanoreactors.

## **Background**

The design of novel hybrid nanostructures with specific functionalities and highly controlled dimensions is among the desired properties of many emerging applications. Recipe development, how to grow nanostructures in specific locations with a certain morphology and functionality, is a challenge for the continuous progress of nanotechnology. In particular, the fabrication of these nanostructures in arrays is an essential key for many applications. From the experimental point of view, it is only recently that the preparation techniques have become available to allow fabrication of these types of materials with a reasonably high degree of control. Nanoporous oxide membranes present a novel class of materials that offer a high potential for a variety of applications [1]. Macroscopic areas of nanopatterned alumina pores can be synthesized, converting these materials into ideal masks for making highly reproducible arrays of nanostructures. One of the major advantages in the use of nanoporous alumina is the cost-efficient synthesis of a large area of nanoporous arrays with well-controlled properties down to nanometer. Some examples of nanoscale patterning using these membranes as a template include the synthesis of semiconducting and metallic dots [2,3], nanowires [4,5], nanotubes and multilayered cylindrical structures [6,7], among other structures [8]. One of the problems in the use of anodized aluminum oxide (AAO) membranes, generally prepared by the anodization of self-supporting (relatively thick) aluminum films [9], is that the AAO membranes cannot be easily incorporated on devices since this step requires the transfer of the membranes onto a substrate. Insufficient adhesion between the membrane and substrate, mask corrugation, and general transfer problems reduce the reproducibility and uniformity of the fabricated structures. In order to avoid these problems, it is possible to fabricate the membranes directly on a substrate by anodization of aluminum films previously deposited on the substrates [3]. On the other hand, it is well demonstrated that carbon nanotubes (CNTs) can be grown inside of these membranes adopting the shape of the pores [10-13]. By tuning the experimental conditions, this method allows to have a high degree of control of the external and internal diameters of the tubes. This possibility, together with the possibility that some properties of the tube can be modified by functionalization or by proximity with dissimilar materials, makes this system an ideal candidate for many applications [14].

In this work, we present the synthesis of palladium and TiO_2_ nanostructures inside carbon nanotubes using these AAO membranes as nanoreactors. We have explored the use of two types of AAO membranes: one was prepared by the anodization of an aluminum foil to obtain a self-supported material (50-μm thick), and the other one prepared by the anodization of a thin aluminum film directly deposited on top of a silicon wafer by e-beam evaporation (approximately 5-μm thick). Depending on the potential application, it is possible to choose between silicon-supported or self-supported membranes. Multi-walled CNTs were grown inside the membranes by decomposition of acetylene without using a metal catalyst [10-13]. This happens since the carbon atoms/fragments from the decomposition of the carbon source nucleate inside the pores adopting its morphology. Since outside walls of the tubes are initially completely covered by the template, we can easily access to the inner cavity of tubes by vapor molecules or metal precursors in liquid dissolutions while the outside wall remains free of any molecules or particles.

## **Methods**

### **Synthesis of porous alumina**

In order to fabricate porous alumina over silicon substrates (AAO/Si), we deposit 5 μm of aluminum (99.999% purity) over a polished *n*-type Si(100) wafers (1 to 10 Ω·cm) by electron beam evaporation. During evaporation, the chamber pressure was kept below 2 × 10^−6^ Torr (base pressure is approximately 2 × 10^−7^ Torr). An evaporation rate of 0.2 nm/s was maintained throughout the evaporation and monitored by a quartz crystal microbalance. The first anodization was performed at 40 V in 0.3 M oxalic acid as electrolyte solution for a period of 3 h. The temperature of the electrolyte was controlled using a water cooler and kept at 5°C. To improve pore regularity and to control the thickness of the final porous masks, we have anodized the aluminum film in two steps [3]. After the first anodization, we employed an aqueous solution with 6.0 wt.% phosphoric acid and 1.8 wt.% chromic acid at 60°C, in order to selectively remove a disordered porous alumina layer and leave an ordered pattern of pore nucleus. The second anodization (0.3 to 2 h) was run under the same conditions, yielding homogeneous and highly ordered membranes with a thickness between 1 to 5 μm depending on the anodization time. The pores were widened with a 5-wt.% phosphoric acid etching at room temperature for 50 min. This etching removes the alumina barrier layer at the pore bottom without affecting the membrane order.

The self-supported porous alumina membranes were prepared from a 99.99% aluminum foil (0.13-mm thickness, Sigma-Aldrich Corporation, St. Louis, MO, USA) by the two-step anodization technique. First, the aluminum foil was washed with detergent and then successively with acetone and water. After that, the aluminum sheets were annealed at 350°C in air for 1 h, followed by etching with a 5% *w*/*w* NaOH solution and then with a diluted solution of nitric acid. Subsequently, the samples were mechanically polished with alumina (0.30 and 0.05 μm mesh), followed by 1 min of electropolishing cycle at 15 V in a 40% H_2_SO_4_, 59% H_3_PO_4_, and 1% glycerine bath. After this treatment, the samples were submitted to a first anodization at 40 V for 6 h in a 0.3-M oxalic acid solution at 20°C. The anodized layers were etched with a 5% H_3_PO_4_ and 1.8% H_2_Cr_2_O_4_ solution at room temperature for 12 h. An ordered pore arrangement was achieved with the second anodization step, performed under the same conditions as the first one. A 0.10-M CuCl_2_/20% HCl solution at room temperature was used to remove the remaining aluminum, in order to obtain a self-supported porous alumina membrane with a thickness close to 50 μm. To remove the barrier layer and open the pores at the bottom, the membranes were treated with 5% H_3_PO_4_ aqueous solution at room temperature. Subsequently, the pores were widened in a 0.085-M H_3_PO_4_ solution at 37°C for 15 min.

### **Synthesis of carbon nanotubes inside of a porous alumina template**

CNTs were synthesized by the decomposition of acetylene inside the pores of AAO that act as templates. The growth of CNTs was performed using a chemical vapor deposition (CVD) apparatus composed of a tube furnace, gas flow lines, digital mass-flow controllers, and a quartz tube as reactor. In a typical synthesis, performed at atmospheric pressure, small pieces of AAO (50 μm) or AAO (1 μm)/Si membrane (approximately 0.5 cm^2^) were put in a quartz boat inside the CVD reactor and heated at a rate of 20°C/min under an Ar stream (100 sccm) until the desired synthesis temperature (650°C) is reached. The synthesis was performed at such temperature with a mixture of Argon/acetylene (200/25 sccm) by periods of 10 to 60 min. Next, the system was cooled down to room temperature under Ar atmosphere. After this process, the obtained samples exhibited a black surface due to carbon formation.

### **Synthesis of carbon nanotubes filled with palladium nanoparticles**

In order to prepare the composites, we have used PdCl_2_ and Pd(NO_3_)_2_ dissolutions. To introduce these precursors into the CNT-AAO membranes, we have employed wet impregnation and incipient wetness impregnation. In these experiments, we have used self-supported AAO membranes (approximately 50-μm thick). The solutions were prepared as follows: for the chloride precursor, 0.49 g of PdCl_2_ + 0.5 g of KCl were diluted in 50 mL of deionized H_2_O in order to form the soluble [PdCl_4_]^2−^ complex; for the nitrate precursor 0.111 g of Pd(NO_3_)_2_·H_2_O was diluted in 10 mL of 2-propanol. To introduce the palladium precursors into the CNT-AAO by wet impregnation, we have immersed approximately 1-cm^2^ pieces of these membranes into the solutions for periods of 24, 72, and 116 h. On the other hand, the incipient wetness impregnation was carried out by solution drop casting into small pieces of these membranes (approximately 12 mm^2^) using 10 and 30 μL for each side of these porous substrates. After impregnation, the membranes were calcinated (350°C) in an O_2_/Ar mixture for 1 h and were reduced (450°C) in H_2_/Ar atmosphere for 1 h.

### **Synthesis of carbon nanotubes filled with TiO**_**2**_

TiO_2_-CNT composites were prepared by CVD of titanium tetraisopropoxide (TTIP) over CNT-AAO-Si substrates. TTIP was introduced to the tube furnace by bubbling Argon (100 to 200 sccm) through a vessel with the titanium precursor previously thermalized at 100°C in a glycerine bath and then was decomposed at 400°C or 500°C. The TTIP precursor has been used by other authors under similar conditions to obtain TiO_2_ deposits [15]. The TiO_2_-CNT hybrids were released from the substrates using the same methodology as that of the palladium composites.

### **Characterization of products**

The anodized aluminum oxide membranes were characterized mainly by scanning electron microscopy (SEM). In order to characterize the formed nanostructures, the AAOs were finally removed using 5% NaOH/water solution, leaving behind nanotubes and nanotubes filled with palladium or titanium dioxide. Next, the products were purified by successive ultracentrifugation processes and by change of solvent to remove the dissolved aluminum oxide. The obtained products were deposited on carbon-coated copper grids to be analyzed by transmission electron microscopy (TEM) and also on silicon substrates to be analyzed by Raman spectroscopy and energy-dispersive X-ray spectroscopy (EDX).

## **Results and discussion**

**Figure 1:**
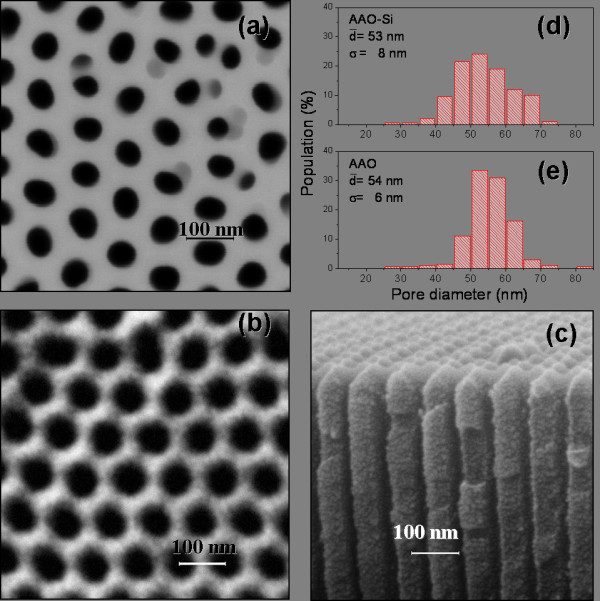
**SEM images of silicon-supported and self-supported AAO membranes.** (**a**) SEM image of the surface of silicon-supported AAO membrane. (**b,c**) SEM images of top and side views of a self-supported AAO membrane, respectively. (**d,e**) Pore diameter distribution for both samples, silicon-supported and self-supported membranes, respectively.

After a two-step anodization process, in aluminum foils or films, it is possible to obtain fairly well-ordered membranes. Figure 1 shows a set of images corresponding to alumina membranes. Figure 1a shows a SEM image of the surface of the porous alumina grown over a silicon substrate. Figure 1b,c shows SEM images of top and side views of a self-supported porous alumina membrane, respectively. From these micrographs, it is possible to observe that silicon-supported and self-supported membranes exhibit a high grade of order especially in the regularity of pores and hexagonal distribution as in the perpendicularity to the substrate (Figure 1c). If we compare both membranes, it is possible to observe that self-supported membranes exhibit a high degree of order. Figure 1d,e shows the pore diameter distribution for both samples, silicon-supported and self-supported, respectively. The silicon-supported membrane has a mean diameter close to 53 nm, whereas the self-supported membrane exhibits a similar diameter of 54 nm, but this last one has a sharper diameter distribution and a lower standard deviation. This difference comes from the fact that for the preparation of silicon-supported membranes, we employ a lower time in the first anodization step. The first anodization is responsible for the formation of pore nucleus; for longer periods, the order increases. Because silicon-supported membranes are prepared from very thin aluminum films, we have a limited thickness, and not longer anodization periods can be used since this implies the total anodization of the aluminum. This fact finally affects the order of the membrane.

**Figure 2:**
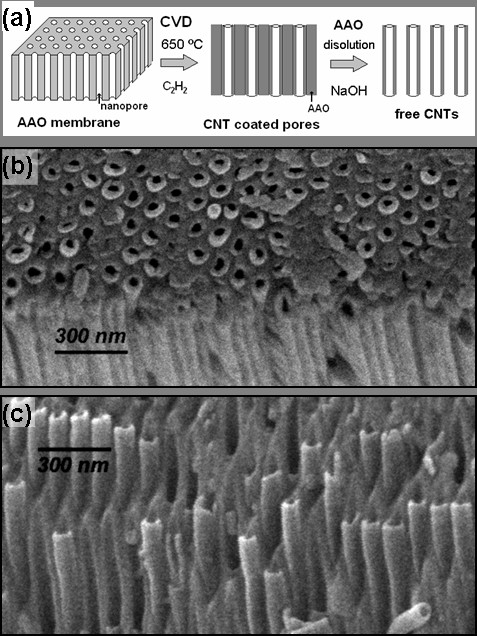
**Synthesis scheme and SEM micrographs of CNTs.** (**a**) Schematic representation of the CNT synthesis steps, (**b**) SEM micrograph of 30-min-grown CNTs inside an AAO template, and (**c**) SEM micrograph showing CNTs after partial dissolution of AAO by NaOH.

Both silicon-supported and self-supported membranes were used to grow CNTs and as templates in the CVD decomposition of acetylene. Figure 2a shows a diagram of the formation of CNTs inside the AAO membranes. Figure 2b corresponds to a SEM micrograph showing the surface of the AAO membranes after the CNTs have grown. CNTs occupy almost all the pores of the membrane, and their external diameter depends only on the AAO pore diameter. The tube lengths do not dependent on the synthesis time since they do not grow beyond the membrane thickness; they are limited by the extension of the pores. Figure 2c shows the AAO-CNT membrane but with the alumina partially dissolved. From the exposed tips of the CNTs, it is possible to note that their growth is fairly well ordered inside the pores following the template array.

**Figure 3:**
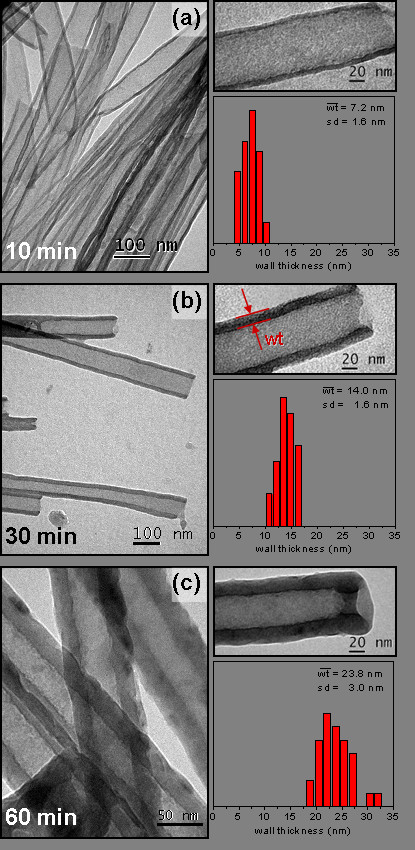
**TEM micrographs and variation of the CNT wall thickness.** Micrographs corresponding to different synthesis times: (**a**) 10, (**b**) 30, and (**c**) 60 min.

Figure 3 shows TEM images of the CNTs prepared using self-supported membrane at different synthesis times (10, 30, and 60 min). Those tubes grow by adopting the inverse morphology of the pores; if the pores have regular sizes, then they also grow regularly, and the resulting tubes always preserve the dimension of the pore. On the other hand, if we change the time of carbon deposition, the tubes grow with a thicker wall, making the inner diameter smaller. Figure 3 also shows the histograms of the wall thickness of these nanotubes. It is possible to observe how the wall thickness increases almost linearly from 7 nm to as close as 24 nm when the synthesis time changes from 10 to 60 min.

**Figure 4:**
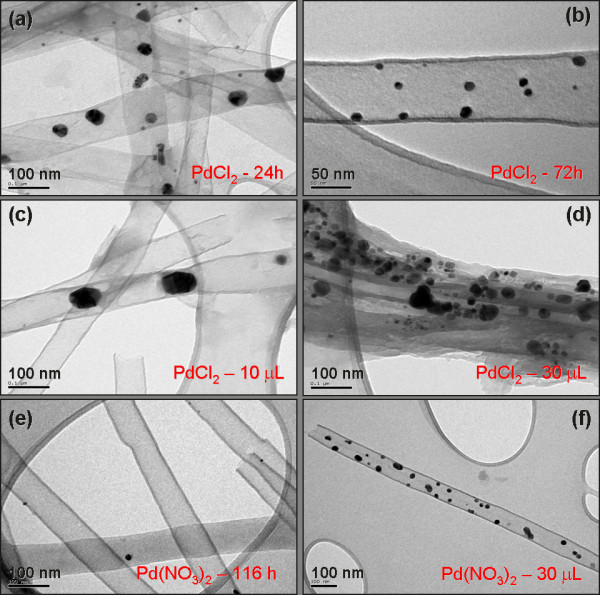
**TEM images of Pd@CNT nanohybrids.** These nanohybrids were prepared using palladium chloride and nitrate precursors by wet impregnation (**a**, **b**, **d**) and incipient wetness impregnation (**c**, **d**, **f**).

Figure 4 shows a set of TEM images of the Pd@CNT hybrid nanostructures prepared using chloride and nitrate palladium precursors. Figure 4a,b shows the hybrids prepared by wet impregnation of CNT (30 min) and AAO membranes with the palladium chloride solution by periods of 24 and 72 h, respectively. Figure 4c,d shows the hybrids prepared by drop casting 10 and 30 μL of palladium chloride solution directly on the CNT (30 min) and AAO membranes, respectively. On the other hand, Figures 4e,f shows the samples prepared using palladium nitrate solution, immersing the CNT-AAO membrane for 116 h and by casting 30 μL of the same solution directly on a piece of CNT-AAO, respectively.

**Figure 5:**
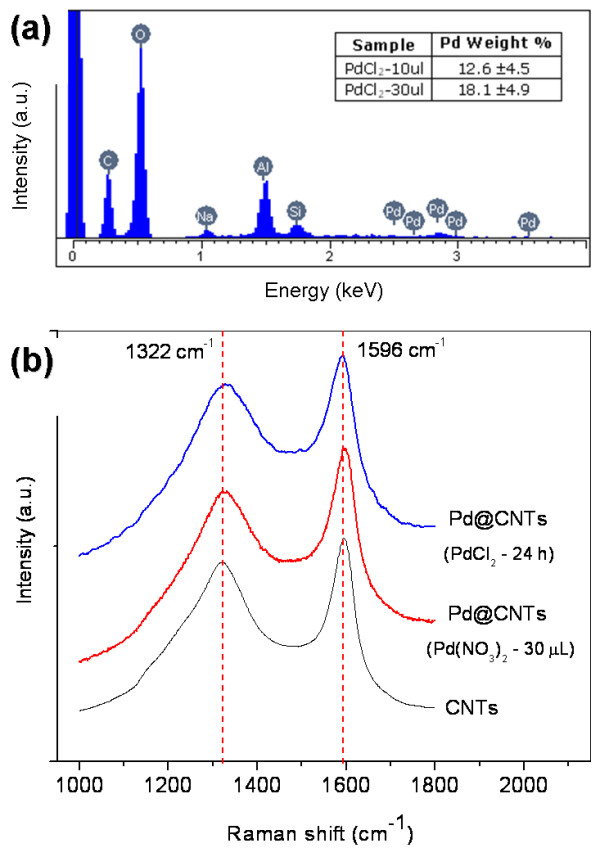
EDX (a) and Raman spectra (b) of Pd@CNT nanohybrids prepared using palladium chloride and nitrate precursors.

Comparatively, it is possible to note from these pictures that the palladium chloride precursor was introduced more efficiently than the palladium nitrate precursor since more particles were formed in the inner cavity of the nanotubes. For example, when we use the nitrate precursor in wet impregnation for periods as long as 116 h, almost no particles were found inside the tubes as compared with those shorter periods using chloride precursor. This exhibits an appreciable amount of palladium nanoparticles inside CNTs. This fact could be associated to the formation of a very soluble metal complex, the [PdCl_4_]^2−^, which could penetrate into the pores more easily than the palladium nitrate precursor which tends to precipitate in dissolution. Additionally, it is possible to observe that the spontaneous diffusion of ions (by wet impregnation) is less efficient in the introduction of palladium inside the tubes. An EDX analysis of those samples supports the TEM observations. These results show that only samples prepared with the chloride precursor exhibit a quantifiable amount of palladium using this technique. Figure 5a shows a typical EDX spectrum of the sample with the higher amount of palladium and an inserted table with the quantification for the hybrids prepared with the chloride precursor by the drop casting technique.

Figure 5b shows the Raman spectra of Pd@CNT samples prepared under different conditions and also the spectrum of pure CNTs. All spectra exhibit characteristic peaks observed in these kinds of CNTs [16]. The *G*-band generated by the E_2g_ vibrational mode and the -band associated to an active A_1g_ breathing mode of the six-member ring are both linked to vibrational modes of sp^2^-bonded carbon atoms. For all our samples, including the pure CNTs, these vibrational modes appear in the same frequency: close to 1,596 cm^−1^ for the *G*-band and 1,322 cm^−1^ for the *D*-band. No significant shifts in these bands were appreciated for the Pd@CNT hybrids with respect to the pure CNT spectrum. Additionally, no variations were detected in the *I*(*G*)/*I*(*D*) ratio; this value was very close to 1.2 for all samples. These results indicate that the introduction of palladium nanoparticles inside the nanotubes does not cause appreciable damage or structural changes in the graphitic material.

**Figure 6:**
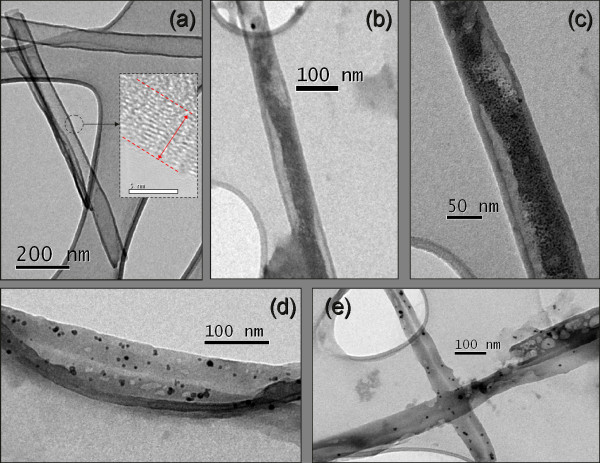
**TEM images of pure CNTs and TiO**_**2**_**@CNT nanohybrids.** TEM images of (**a**) pure CNTs and TiO_2_@CNT nanohybrids (b to e) prepared by CVD of TTIP precursor using AAO-Si substrates. The insert in (a) shows an HRTEM image of the wall structure of a pure CNT. (**b**, **c**) Images of samples prepared at 500°C and 200 sccm of Ar. (**d**, **e**) Image of samples prepared at 400°C and 100 sccm.

In order to probe the use of CNT-AAO membranes with vapor phase precursor, we have explored the use of titanium isopropoxide to form TiO_2_ inside CNTs. Figure 6 shows TEM images of CNTs and TiO_2_@CNT hybrid composites prepared using the silicon-supported membrane. Figure 6a shows an image of a pure CNT sample after release from the AAO. The insert of Figure 6a displays a high resolution image of the wall structure of CNTs wherein it is possible to observe that the tubes synthesized are of a multi-walled nature with a relatively high degree of disorder, as we can also observe in the Raman spectrum of pure CNTs prepared from a self-supported membrane (Figure 5b). Figure 6b,c exhibits micrographs of the samples prepared at 500°C, 200 sccm of Ar, whereas Figure 6d,e shows the micrographs of the samples prepared at 400°C and 100 sccm. When we compare these images with the image of pure CNTs (Figure 6a), the results show that TiO_2_ effectively covered the inner cavity of carbon nanotubes since an appreciable amount of material was deposited inside the cavity. Different variables have shown that the morphology of the deposit can be changed; for example, the deposits prepared at 500°C look more like a film inside the nanotubes as compared with the samples prepared at 400°C that exhibit a distribution of small nanoparticles embedded in a film.

## **Conclusions**

The results obtained have shown that the pores of an anodized aluminum oxide can be easily used to prepare carbon nanotubes inside them, as other researchers have also reported [10-13,17]. Additionally, we have demonstrated that nanotubes prepared inside AAO pores can be used as nanoreactors for the growth of diverse material nanostructures. These preliminary results show that AAO-CNT membranes are very useful templates to prepare metallic or semiconductor nanostructures selectively inside CNTs; differences of other methods employed by our group yielded nanoparticles deposited outside of nanotubes [18,19]. These facts are possible since the outer wall of CNTs is protected by the AAO membrane, and the inner cavity is open, allowing the diffusion of material precursors dissolved in liquids and also in vapor phase. Once the nanostructures have been formed inside the nanotubes, by impregnation/calcinations/reduction in the case of palladium and by chemical vapor deposition/decomposition in the case of TiO_2_, the AAO can be easily removed to release the hybrid nanotube-based nanostructures. Depending on the potential applications, both the silicon-supported and self-supported membranes can be used indistinctly to prepare these kinds of hybrid nanostructures, but the silicon-supported membrane is more suitable for the integrated devices.

## Abbreviations

AAO, anodized aluminum oxide; CNT, carbon nanotube; CVD, chemical vapor deposition; SEM, scanning electron microscopy; TEM, transmission electron microscopy; TTIP, titanium (IV) tetraisopropoxide.

## **Competing interests**

The authors declare that they have no competing interests.

## **Authors’ contributions**

The work presented here was carried out in collaboration among all authors. RS and SH defined the research theme. VN, CC, JV, AC and PH carried out the laboratory experiments under RS and SH supervision. RS performed TEM, and SH performed the SEM and Raman measurements. RS and SH have discussed the results and wrote the manuscript. All authors read and approved the final manuscript.
